# Chromosome-level genome assembly of a high-altitude-adapted frog (*Rana kukunoris*) from the Tibetan plateau provides insight into amphibian genome evolution and adaptation

**DOI:** 10.1186/s12983-022-00482-9

**Published:** 2023-01-06

**Authors:** Wei Chen, Hongzhou Chen, Jiahong Liao, Min Tang, Haifen Qin, Zhenkun Zhao, Xueyan Liu, Yanfang Wu, Lichun Jiang, Lixia Zhang, Bohao Fang, Xueyun Feng, Baowei Zhang, Kerry Reid, Juha Merilä

**Affiliations:** 1 School of Resources and Environmental Engineering, Anhui University, Hefei, 230601 China; 2 Anhui Shengjin Lake Wetland Ecology National Long-Term Scientific Research Base, Dongzhi, 247230 China; 3 Anhui Province Key Laboratory of Wetland Ecosystem Protection and Restoration, Anhui University, Hefei, 230601 China; 4 School of Life Science and Technology, Mianyang Normal University, Mianyang, 621000 Sichuan China; 5 Department of Ecology, College of Life Sciences, Henan Normal University, Xinxiang, 453007 China; 6 Department of Organismic and Evolutionary Biology and Museum of Comparative Zoology, Harvard University, 26 Oxford Street, Cambridge, MA USA; 7 Ecological Genetics Research Unit, Research Programme in Organismal and Evolutionary Biology, Faculty of Biological and Environmental Sciences, University of Helsinki, 00014 Helsinki, Finland; 8 School of Life Sciences, Anhui University, Hefei, 230601 China; 9 Area of Ecology and Biodiversity, School of Biological Sciences, The University of Hong Kong, Hong Kong SAR, China

**Keywords:** Chromosome, High-altitude adaptation, Genome, Hi-C sequencing, Illumina, PacBio sequencing, *Rana kukunoris*

## Abstract

**Background:**

The high-altitude-adapted frog *Rana kukunoris*, occurring on the Tibetan plateau, is an excellent model to study life history evolution and adaptation to harsh high-altitude environments. However, genomic resources for this species are still underdeveloped constraining attempts to investigate the underpinnings of adaptation.

**Results:**

The *R. kukunoris* genome was assembled to a size of 4.83 Gb and the contig N50 was 1.80 Mb. The 6555 contigs were clustered and ordered into 12 pseudo-chromosomes covering ~ 93.07% of the assembled genome. In total, 32,304 genes were functionally annotated. Synteny analysis between the genomes of *R. kukunoris* and a low latitude species *Rana temporaria* showed a high degree of chromosome level synteny with one fusion event between chr11 and chr13 forming pseudo-chromosome 11 in *R. kukunoris*. Characterization of features of the *R. kukunoris* genome identified that 61.5% consisted of transposable elements and expansions of gene families related to cell nucleus structure and taste sense were identified. Ninety-five single-copy orthologous genes were identified as being under positive selection and had functions associated with the positive regulation of proteins in the catabolic process and negative regulation of developmental growth. These gene family expansions and positively selected genes indicate regions for further interrogation to understand adaptation to high altitude.

**Conclusions:**

Here, we reported a high-quality chromosome-level genome assembly of a high-altitude amphibian species using a combination of Illumina, PacBio and Hi-C sequencing technologies. This genome assembly provides a valuable resource for subsequent research on *R. kukunoris* genomics and amphibian genome evolution in general.

**Supplementary Information:**

The online version contains supplementary material available at 10.1186/s12983-022-00482-9.

## Background

Recent advances in sequencing techniques have led to the rapid increase in high-quality vertebrate genomes available to the evolutionary biology community [1–3]. However, complete genome assemblies of anurans remain relatively rare due to their large size (> 4 GB, range: 4 GB to more than 8 Gb GB) and complexity due to repetitive elements which makes them expensive to sequence and challenging to assemble [4]. Studies focused on the evolution and ecology of anurans are important due to their sensitivity to environmental changes, but the lack of high-quality genomic resources and large-scale re-sequencing projects is limiting progress towards understanding the genetic underpinnings of their adaptation to new and changing environments [4, 5]. To date, out of ca. 8100 amphibian species described [6], about10 anuran genomes have been published including *Xenopus tropicalis* [7]*, Nanorana parkeri* [5], *Xenopus laevis* [8], *Rana catesbeiana* [9], *Rhinella marina* [10], *Oophaga pumilio* [11], *Leptobrachium leishanense* [2], *Vibrissaphora ailaonica* [12]*, Bufo gargarizans* [3] and *Rana temporaria* [13]. The genomes that are already available provide a great resource for amphibian comparative genomic research, but to advance our understanding of amphibian genome evolution and amphibian adaptation to their current and future habitats, additional genomes are required. In particular, additional genomes from species closely related to already sequenced species but adapted to unique environmental conditions would be of interest to decipher the genomic basis of adaptations.

*Rana kukunoris*, which is known as the Plateau brown frog, is a true frog from the family Ranidae and is an endemic species living in the Qinghai-Tibetan Plateau of China and distributed in altitudes ranging from 2200 to 4400 m [14]. Living at such high altitudes exposes *R. kukunoris* to extreme environmental conditions including hypoxia, high levels of UV-B radiation and dramatic temperature fluctuations [5]. Consequently, *R. kukunoris* provides an excellent biological model to study amphibian adaptations to extreme high-altitude conditions. However, current research has mainly been limited to a few phylogenetic studies which have tried to resolve its status as a valid species [15], larger scale biogeographic studies of Chinese brown frogs [16] and sex-chromosome evolution and turnover in true frogs [17].

The genome of *R. kukunoris* is thought to consist of 2n = 24 chromosomes [18], with *Rana* species in general having 26 diploid chromosomes (n = 13) but chromosome number has been observed vary from 2n = 22 to 2n = 24 [19]. Its mitochondrial genome has been assembled which revealed that *R. kukunoris* is closely related to *R. temporaria* and *R. chensinensis* [20]. Recent genomic studies of this species have focused on generating transcriptomic data to identify differentially expressed genes associated with high altitude [20]. However, having a well assembled, high-quality, chromosome-level genome would facilitate future research to study the genomic underpinnings of adaptation to differing environments and anuran genome evolution in general [21–23].

In this study, we report the assembly and annotation of the complex and large de novo genome of a male *R. kukunoris* using Illumina short-read, PacBio long-range and Hi-C sequencing and comparison of this assembly to that of the ten available anuran genomes. To this end, we assessed whole-genome synteny, transposable elements and their distribution, amphibian-specific highly conserved elements (HCEs), and changes in functionally important gene families among species. This genome will facilitate future in-depth phylogenomic studies, whole-genome resequencing studies, investigations into how species adapt to extreme high-altitude environments and how genomes of amphibians evolve.

## Results

### Genome survey

**Fig. 1:**
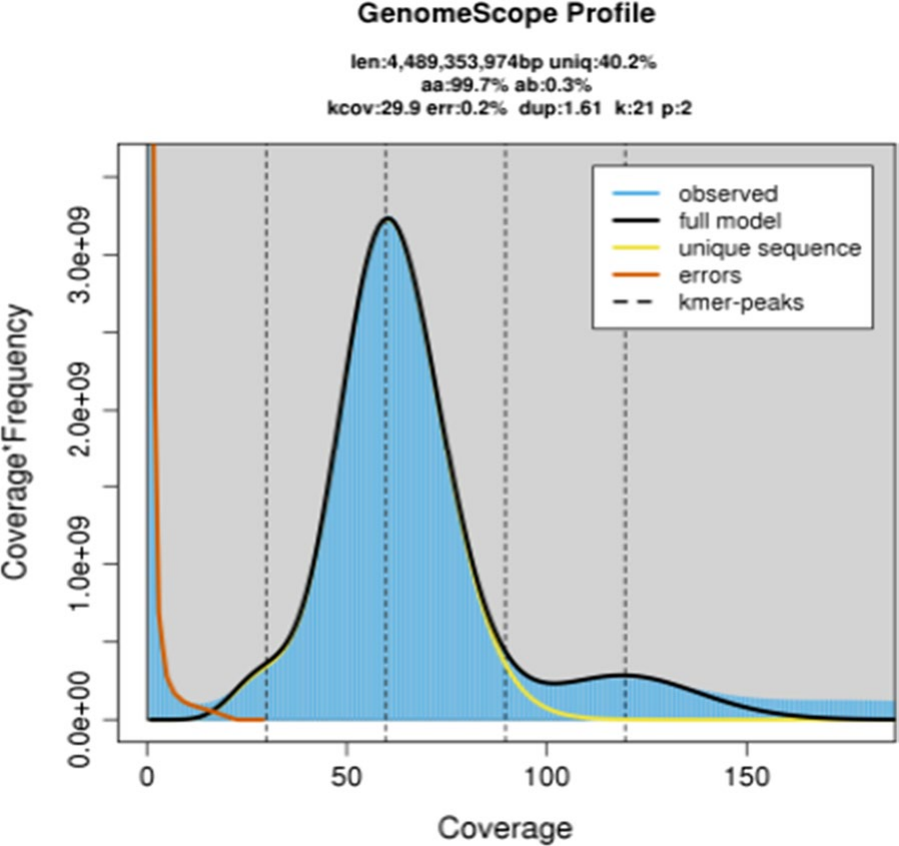
21-mer analysis of *Rana kukunoris* genome characteristics

The Illumina sequencing produced 322.83 Gb of high-quality sequences at a total sequencing depth of 71.91×. The haploid genome size of *R. kukunoris* with 21 k-mer coverage evaluation was estimated to be ~ 4.49 Gb (4,489,353,974 bp, peak = 29, SI Appendix, Additional file 1: Table S2 and Fig. 1) and the estimated heterozygosity rate was ~ 0.3%. Repetitive DNA content was estimated at 59.81% and GC content at 43.62%, suggesting that *R. kukunoris* has a large and complex genome.

### Genome assembly

**Fig. 2:**
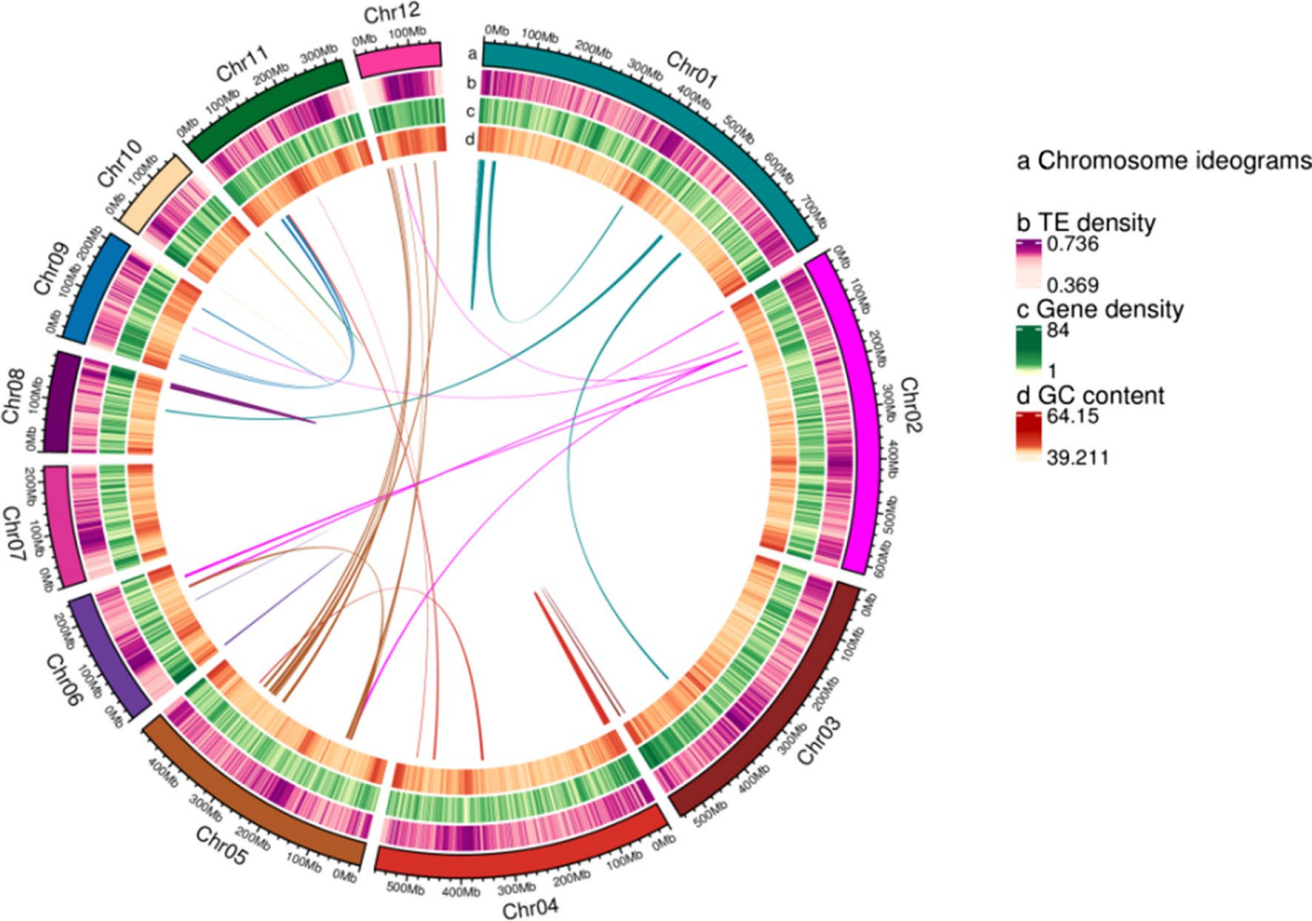
The multidimensional landscape of *Rana kukunoris* genome. Rings, from outside to inside, correspond to: (a) Chromosome ideograms; (b) TE density; (c) gene density and (d) GC content. Lines inside the circle display collinear synteny

PacBio Sequel II sequencing results generated a total of ~ 437.68 Gb (437,678,042,147 bp) sequencing data. The average read length and N50 length of PacBio reads were 20.53 Kb (20,527 bp) and 30.65 Kb (30,654 bp), respectively (Additional file 1: Tables S3, S4), and total sequencing depth was 90.61×. After polishing the assembly using a combination of PacBio and Illumina sequencing data to improve its accuracy, we obtained a genome of ~ 4.83 Gb (4,830,373,361 bp) with contig N50 of ~ 1.80 Mb (1,798,518 bp) and a GC content of 44.41% (Table 1; Additional file 1: Table S5). The contig N50, total genome size and coverage in this assembly were relatively high compared to the amphibian genomes reported to date (Additional file 1: Table S6). The constructed Hi-C libraries produced 1,170,517,199 validated interaction pairs to achieve a chromosome-level *R. kukunoris* genome, being clustered in 6555 scaffolds with the final genome length of ~ 4.81 Gb (4,814,345,922 bp) and grouped into 12 pseudochromosomes (Fig. 2; Additional file 1: Table S7).

**Table 1:** Summary statistics for the *Rana kukunoris* genome

	Assembly
Number of contigs	6555
Total size of contigs	4,830,373,361
Longest contigs	11,379,543
N50 scaffold	547,819,331
GC content (%)	44.41
Assembly validation	
Complete BUSCOs	3096 (92.31%)
Complete single-cope BUSCOs	2945 (87.81%)
Complete duplicated BUSCOs	151 (4.50%)
Fragmented BUSCOs	63 (1.88%)
Missing BUSCOs	195 (5.81%)
Total BUSCOs groups searched	3354

Up to 99.50% of the Illumina raw reads (NCBI Accession No.: SRR17818264-17818270) could be mapped back to the assembled *R. kukunoris* genome (Additional file 1: Table S8), suggesting that most reads could be successfully assembled into the genome. According to the CEGMA database (version 2.5), 93.45% of 458 Core Eukaryotic Conserved Genes (CEGs) were present in the assembly and 72.18% of 248 highly conserved CEGs were present. In addition, ~ 92.31% of the complete BUSCO genes were recovered including 87.81% complete and single-copy BUSCOs(S), 4.50% complete and duplicated BUSCOs (D), 1.88% fragmented BUSCOs(F) and 5.81% missing BUSCOs (M) (Additional file 1: Table S9). In general, the *R. kukunoris* genome showed high genomic integrity.

### Synteny analysis

**Fig. 3:**
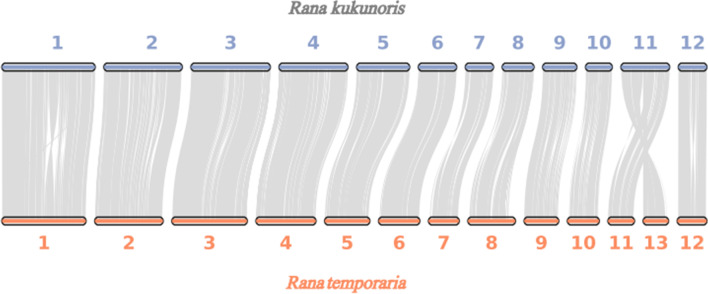
Chromosome synteny between *R. kukunoris* and *R. temporaria genomes*

To explore the genome evolution, synteny analysis was performed between the *R. kukunoris* (high-altitude species) and *R. temporaria* (low-altitude species) genomes. A high degree of collinearity was observed, each chromosome of *R. kukunoris* having corresponding chromosome in *R. temporaria.* For instance, most of the synteny blocks of *R. kukunoris* in the pseudo-chromosomes p-chr1, p-chr2, p-chr3 p-chr4, p-chr5, p-chr6, p-chr7, p-chr8, p-chr9, p-chr10, and p-chr12 were matched to *R. temporaria* chr1, chr2 and chr3, chr4, chr5, chr6, chr7, chr8, chr9, chr10 and chr12, respectively. However, the *R. kukunoris* p-chr11 appeared to be fusion of *R. temporaria* chr11 and chr13, suggesting chromosome fusion/fission events between the two species (Fig. 3).

### Genome annotation

In total, 32,304 protein-coding genes were predicted according to ab initio prediction (Stanke et al., 2006), homology-based prediction and RNA-seq data (NCBI Accession No.: SRR17284458; Additional file 1: Table S9). In total, 31,957 of the 32,304 genes, up to 98.93% of the *R. kukunoris* genes, were annotated across eight databases including KEGG, GO, KOG, SwissProt, Pfam, TrEMBL, eggNOG and NR (Additional file 1: Tables S10, S11). Also, 8554 tRNA, 2510 rRNA, 210 miRNA, 2144 snoRNA and 184 snRNA were predicted. Approximately 61.46% (2,968,809,293/4,830,373,361 bp) of genome appears to consist of TEs, of which retroelements and DNA transposons accounted for 38.60% and 22.85%, respectively (Additional file 1: Table S12). Approximately 9.47% (457,368,139 bp) of the *R. kukunoris* genome was indicated to be tandem repeats (Additional file 1: Table S6). Based on the comparison with 10 other anuran genomes used in this study, there was no correlation neither between genome size and number of protein-coding genes, nor between genome size and the proportion of repetitive elements in the genome across the species (*p* > 0.5 in both comparisons, Additional file 1: Table S5).

### Phylogenetic relationships, evolution and expansion of gene families

**Fig. 4:**
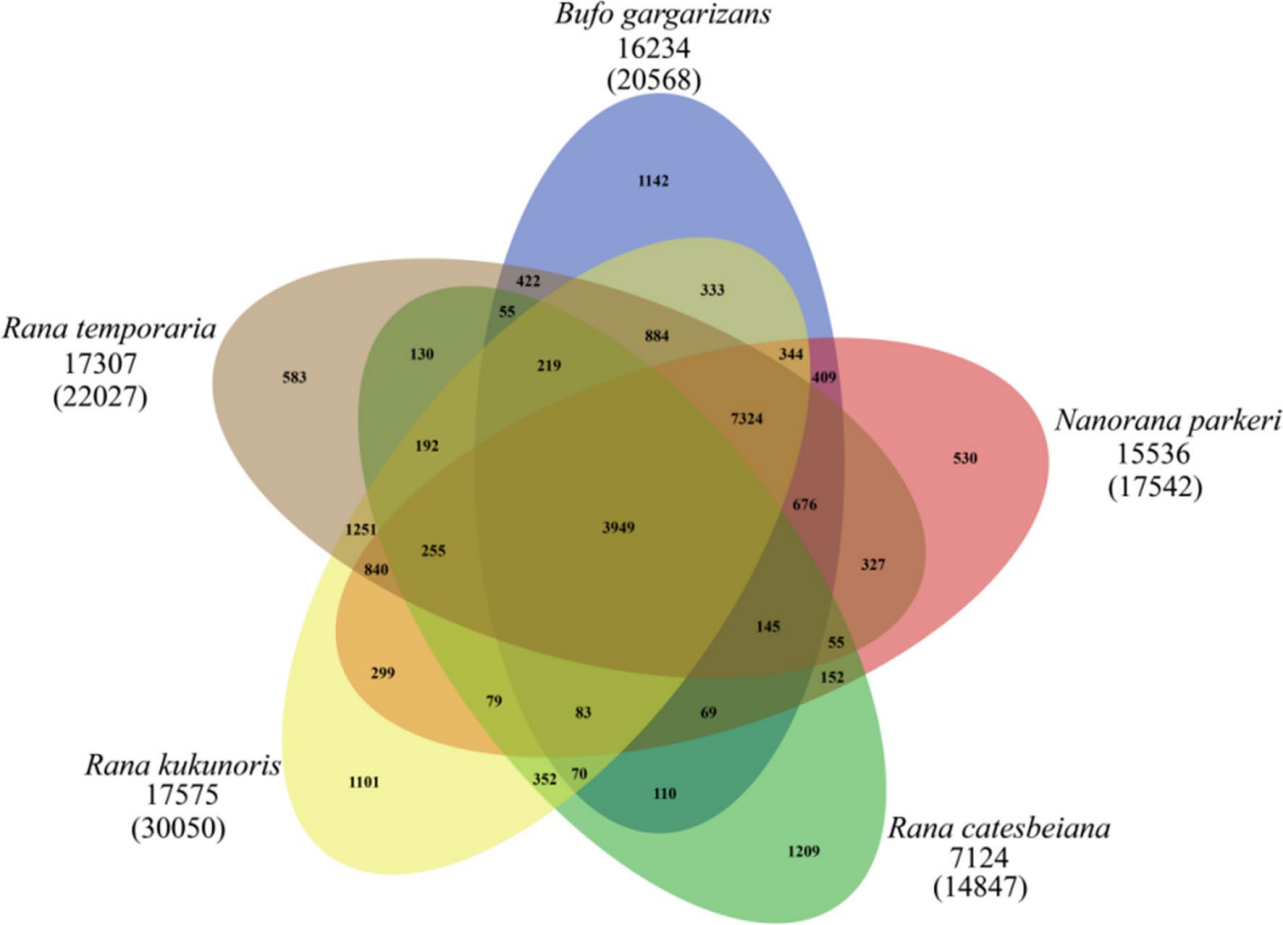
A Venn diagram showing the clustering of gene families among *B. gargarizans*, *N. parkeri*, *R. catesbeianus*, *R. temporaria* and *R. kukunoris*

**Fig. 5:**
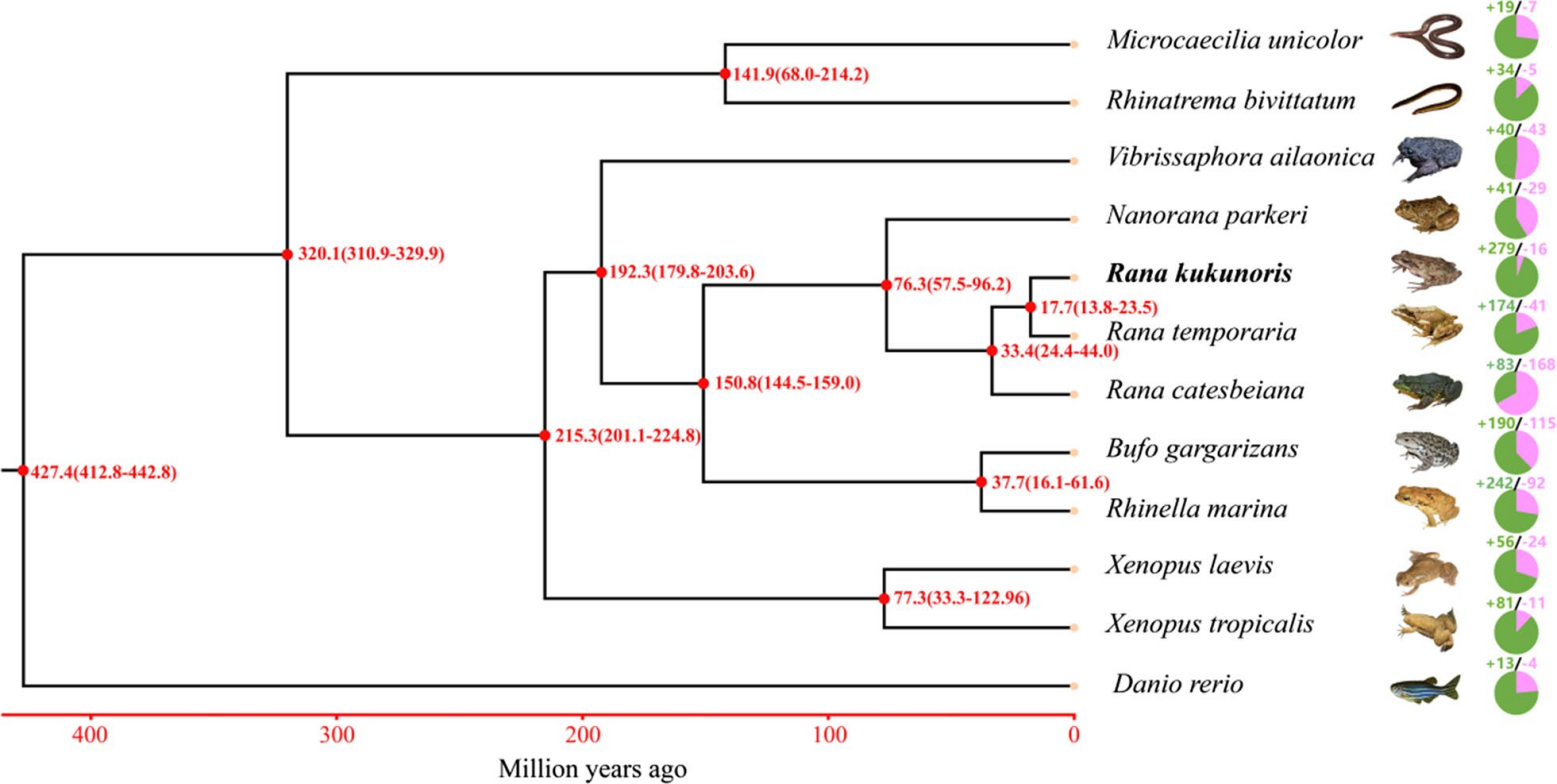
The phylogenetic relationships and estimated divergence times among 11 amphibian species based on 176 single-copy orthologus genes, *Danio rerio* was used an outgroup species. Pie diagrams indicate the expansion and contraction of gene family in different species. Purple = expanded gene families, Green = contracted gene families

**Fig.6:**
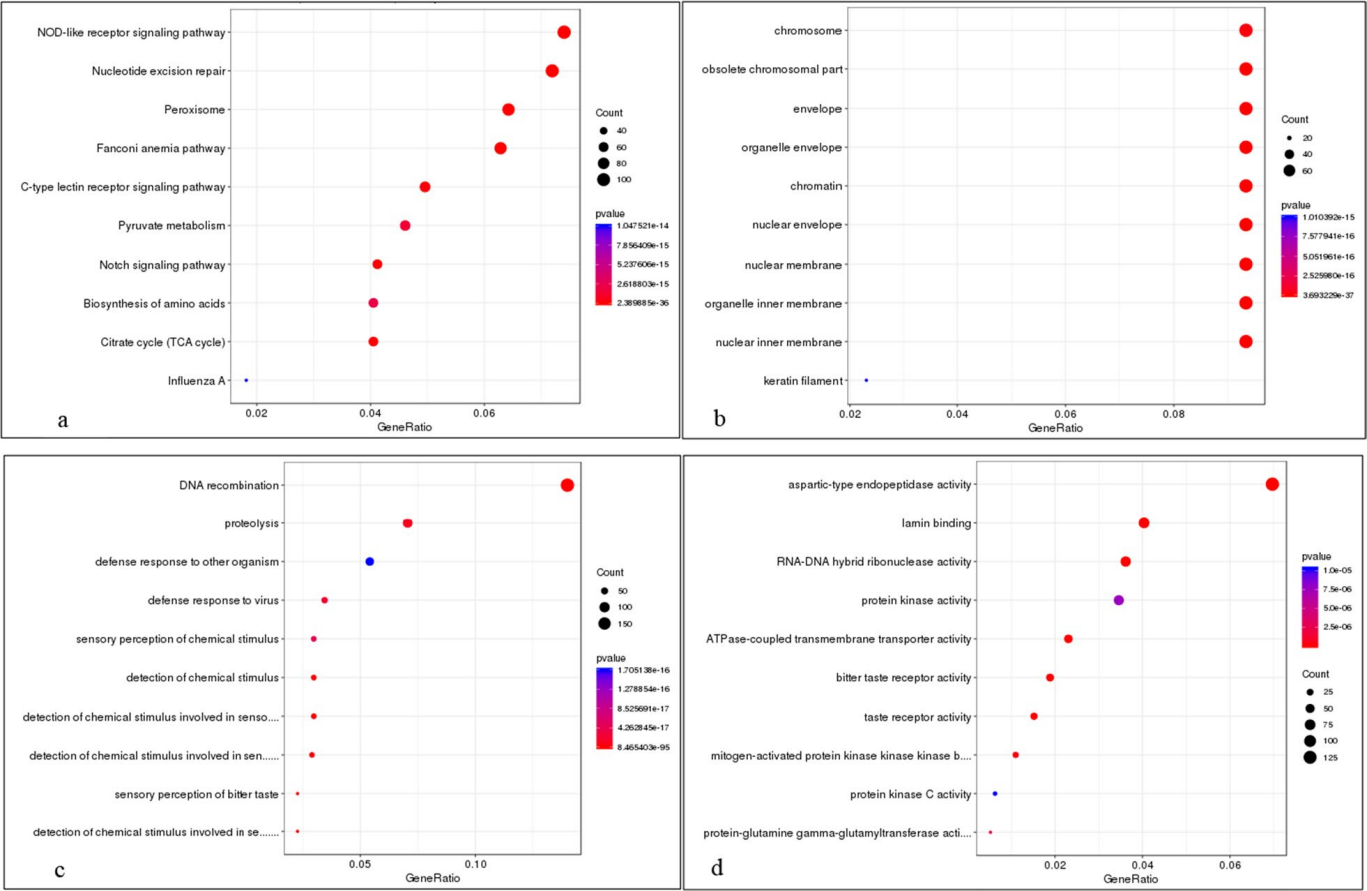
KEGG pathway and functional enrichment analysis of expanded gene families. The size of the dots are indicative of gene counts enriched in the pathway, and the color of the dot shows the significance of the enrichment. **a** Kegg pathway; **b** Cellular component; **c** Biological process; **d** Molecular function

Comparison of the *R. kukunoris* genome with 10 other anuran genomes revealed that 30,050 of the 32,304 protein coding genes belonged to 17,575 orthologous groups (Fig. 4), of which 176 were single-copy orthologous genes. Reconstruction of phylogenetic relationships using these 176 single-copy genes revealed that *R. kukunoris* clustered with *R. temporaria* and *R. catesbeiana* forming their sister lineage (Fig. 5). The estimated divergence time between *R. kukunoris* and *R. temporaria* was 17.7 Ma (13.8–23.5 Ma; Fig. 5). We also identified 279 expanded and 16 contracted gene families in *R. kukunoris* (Fig. 5). Functional enrichment analysis of expanded gene families revealed DNA recombination GO (Gene Ontology) terms that were significantly enriched. The expanded gene families were mainly related to the innate immune system, DNA repair, and taste sense (Additional file 1: Table S13). The KEGG annotation of the expanded genes suggested that the pathway of the NOD-like receptor signaling pathway had the highest ratio, followed by nucleotide excision repair (Fig. 6).

### Functional enrichment and KEGG annotation of the positively selected genes

We detected 97 single-copy orthologous genes having been under positive selection in the *R. kukunoris* genome (Additional file 1: Table S14). The positively selected genes (PSGs) were classified by GO annotation into three different function categories. Cellular component annotations were primarily related to RNA polymerase complex and catalytic complex. Molecular functions were mainly protein domain specific binding, SH3 domain binding and peptidase activity. Biological process annotations were mainly negative regulation of developmental growth, interaction with symbionts and regulation of gluconeogenesis. The results regarding KEGG annotation showed that p53 signaling pathway and FoxO signaling pathway had the highest ratios (Additional file 1: Fig. S1).

## Discussion

Our results showed that *R. kukunoris* has a complex and large genome with a size intermediate (4.83 Gb) to other sequenced *Rana* genomes. Its genome is the third largest of all anuran genomes sequenced so far and like other assembled anuran genomes (Additional file 1: Table S5), over half of the *R. kukunoris* genome consisted of repetitive elements. In size and structure, the *R. kukunoris* genome appears to be similar to that of its close relative, *R. temporaria* [13]. In addition, we also detected several proteins in KEGG pathway of the positively selected genes, which may be associated to the adaptation of high-altitude conditions.

Amphibian genomes contain a lot of repetitive sequences [5]. Similar to the results of previous studies [3, 12], we found that the genome assembly of *R. kukunoris* consisted of 70.91% repetitive DNA with TEs making up 61.46% of its genome. Hence, the large genome of *R. kukunoris* seems to be mainly due to the accumulation of interspersed repeats. To this end, our results are consistent with those of previous studies suggesting that LTR expansions underlie genome gigantism in amphibians [3, 5, 9].

Both ultimate and proximate cause of genome size variation among species remain challenging to understand [24–28]. Our results align with the common finding that species with large genomes do not necessarily have more genes, especially protein-coding genes, than species with smaller genomes [29–31]. Based on the comparison of 11 anuran genomes used in this study, we did not find any correlation neither between genome size and number protein-coding genes, nor between genome size and the proportion of repetitive elements in the genome across the species. One possible reason for latter finding is that the old repeat elements may have accumulated enough mutations so that they are not recognized anymore as repetitive elements.

Hypoxia, high levels of UV-B radiation and dramatic temperature fluctuations are typical characteristics of high-altitude environments [20, 32] and they are believed to be the driving forces of genome evolution of organisms in these habitats [5, 33]. Fluctuating temperatures will impact on energy metabolism and can activate certain amino acid and carbohydrate metabolism related genes such as hpd, ALPL, Aldh6a1, g6pd, PCK2, NADSYN1, Aldh6a1, and G6PD[5, 33–36]. We predicted that these genes would also be important for adaptation of the Tibetan tree frog to its high-altitude habitat, however no direct evidence for positive selection on these genes were found. In addition, melanogenesis (the process of producing melanin pigments) may be important for adaptation to higher UV radiation intensity and is a complex process [34]. However, we did find evidence for positive selection of the creb3l2 protein in the kegg pathway of melanogenesis. nucleotide We did find evidence for positive selection for excision repair (DDB1 protein) which may reflect adaptation to high UV radiation intensity. Furthermore, although we would have predicted EPAS1 to be positively selected in *R. kukunoris* because it has been demonstrated to be under selection for adaptation to hypoxia at high altitudes in Tibetan species [33, 35, 36], we did not detect EPAS1 in KEGG pathway of the positively selected genes.. Likewise, we did not found the FEN1 genes coding for proteins involved in DNA damage repair among the positively selected genes. Taken all this together, the results indicate that different genetic mechanisms may underly different plateau living organisms’ adaptation to their environment [5, 33]. Finally, we note that the abovementioned genes and/or proteins should be the focus of future studies and further functional validation.

## Conclusions

In conclusion, we have described a high-quality genome assembly of *R. kukunoris* which is the 34^th^ amphibian genome assembled (17^th^ chromosome-level genome assembly) so far and the first chromosome-level genome assembly from a high-altitude species. This chromosome-level genome assembly should provide a valuable comparable genomic resource for studies of amphibian genome evolution particularly because it provides opportunity to focus on amphibian genome evolution at much shorter timescales than has been previously possible (cf. Fig. 5). The assembly also provides a resource for population genomic studies of this species, aiding attempts to uncover genomic underpinnings of their adaptation to high-altitude environments.

## Methods

### Sample collection

An adult male of *R. kukunoris* was collected from Yushu, western China (32° 32′ 8.35″ N, 96° 25′ 0.63″ E, 4000 m a.s.l). To obtain sufficient high-quality DNA for the PacBio Sequel II platform (Pacific Biosciences, USA) and Illumina Novaseq 6000, the frog was euthanized with MS-222 and dissected (Ethical proof No. IACUC(AHU)-2022-007). Fresh tissues were retrieved and were snap-frozen in liquid nitrogen for 10 min, and then delivered to the sequencing company on dry ice (the Biomarker Technologies Company, Qingdao, China). DNA was extracted from the muscle and liver tissues. To aid the protein-coding gene annotation of *R. kukunoris*, RNA was extracted from the brain, heart, testis, stomach, intestines, fat, lungs, and skin tissues for transcriptome sequencing.

### Illumina and PacBio sequencing

Extracted DNA was sequenced using the Illumina Novaseq 6000 and PacBio Sequel II platforms and extracted RNA was sequenced using the Illumina Novaseq 6000. The PacBio long-reads were used for genome assembly with Smartdenovo software (version 1.06) (https://github.com/ruanjue/smartdenovo), whereas Illumina short reads were used to estimate genome size and to correct errors in the assembled genome. Library preparations of Illumina and PacBio sequencing were conducted following the manufacturer’s protocols. In brief, for the Illumina sequencing, DNA was sonicated to a fragment size of 350 bp, then three libraries were built by terminal repairing, poly-A and adaptor adding, target fragment selection and PCR amplification. To generate PacBio libraries, DNA was sheared using a Covaris g-Tube and a SMRT-bell library was prepared using the SMRTbellR Express Template Preparation Kit (PacBio DNA template prep kit 2.0; 20–30 Kb). Hi-C libraries were constructed using the NEBNext® Ultra™ RNA Library Prep Kit (NEB, UK) according to the manufacturer’s instructions and sequenced using the Illumina NovaSeq 6000 to anchor scaffolds and facilitate genome annotation. Illumina RNA-seq libraries were also prepared following the manufacturer’s instructions and extracted RNA was pool-sequenced using the Illumina NovaSeq 6000 with a read length of PE150. All sequencing was performed by the Biomarker Technologies Company (Qingdao, China).

### Genomic data processing and genome survey

Adapters were removed from the sequence reads and low-quality reads were filtered out using fastp (parameters: -q 10 -u 50 -y -g -Y 10 -e 20 -l 100 -b 150 -B 150) [37]. PCR duplicates were removed with samtools (version 1.9) [38]. The filtered reads were used to estimate genome size and other characteristics including heterozygosity, repetitive DNA content and GC content. The k-mer distribution was estimated using the jellyfish software (version 2.1.4) with parameter -h 1,000,000,000 [39]. The heterozygosity ratio of the genome and genome size were estimated using GenomeScope (version 2.0) with the following parameters -k 21 -p 2 -m 100,000 [40]. We followed the rule of best k-mer selection: 4 K-mer ≥ genome size [41]. As this is 4^21^ ≈ 4398 Gb, the appropriate selected k-mer size chosen was 21-mers. The genome size was estimated using the following modified formula: G = Nk mer/Daverage k-mer, where G is genome size, Nk-mer the total number of k-mers, Daverage k-mer the average depth of k-mers [42].

### Genome assembly

The raw data from PacBio SequelII platform was corrected with Canu (version 1.5) [43] and the error-corrected PacBio long reads were de novo assembled with Smartdenovo version 1.0 (https://github.com/ruanjue/smartdenovo). To further improve the accuracy of the assembly, the final assembled genome sequence was polished by pilon (version 1.18) based on Illumina raw data with default parameters [44]. To evaluate the accuracy of the assembled genomic sequence, the Illumina sequencing data (NCBI Accession No.: SRR17818264-17818270) was mapped back to the assembled genome using bwa (version 0.7.12) with default parameters [45]. To evaluate the completeness of the genome assembly, Benchmarking Universal Single-Copy Orthologs (BUSCO) (parameters: –evalue 1e−03 (E-value cutoff for BLAST searches), -sp human) was used to search for annotated genes in the assembled genome based on the vertebrata_odb9 database [46]. In addition, the assembly was also investigated with the Core Eukaryotic Genes Mapping Approach (CEGMA version: 2.5) with default parameters [47].

To anchor scaffolds into pseudo-chromosomes, adapter sequences and low-quality raw reads were filtered out using fastp (parameters: -q 10 -u 50 -y -g -Y 10 -e 20 -l 100 -b 150 -B 150). After this, the clean paired-end reads were mapped back to the draft assembly with bwa aligner (version: 0.7.10-r789) and default parameter settings to obtain the unique mapped paired-end reads [48]. Only uniquely mapped reads with a mapping quality > 20 were retained for further analysis. Invalid read pairs, including dangling-end and self-cycle, re-ligation and dumped products, were filtered by HiC-Pro (version 2.10.0) [49]. The uniquely mapped read pairs were used for correction of scaffolds by clustering, ordering and orientating scaffolds into chromosomes by the LACHESIS de novo assembly pipeline (version 1.0) [50] using the parameters: CLUSTER_MIN_RE_SITES = 94; CLUSTER_MAX_LINK_DENSITY = 2; ORDER_MIN_N_RES_IN_TRUNK = 51; ORDER_MIN_N_RES_IN_SHREDS = 51.

### Synteny analysis

To determine genetic differences between low-altitude (*R. temporaria*) and high-altitude species (*R. kukunoris*), the assembled chromosomes of *R. kukunoris* were aligned to the well-assembled chromosomes of *R. temporaria* (accession no. GCF_007399415.2) using Diamond (version 0.9.29.130) with the following parameters e < 1e−5 and C score > 0.5 [51] and the collinearity blocks between species were investigated using MCScanX [52]. After filtering the aligned blocks shorter than 2 Mb in length, we plotted the results using circos [53] and JCVI (version 0.9.13) [54].

### Gene prediction and annotation

Three approaches (de novo prediction, homology search, and transcript-based assembly, respectively) were integrated to predict protein-coding genes in the *R. kukunoris* genome. The de novo gene models were predicted using two ab initio gene-prediction software tools, including Augustus (version 2.4) with the default parameters [55] and SNAP (version 2006-07-28)[56]. For the homolog-based approach, GeMoMa (version 1.7) [57] was performed using the reference gene model from seven species including *B. gargarizans, N. parkeri, R. catesbeianus, R. marina, R. temporaria, X. laevis,* and *X. tropicalis* (Additional file 1: Table S1). For the transcript-based prediction, Trinity software (version 2.11) was used to assemble genes [58] and the PASA software (version 2.0.2) [59] was used to predict genes. Gene models from these different approaches were combined using the EVM software (version 1.1.1) [60] and updated by PASA (version 2.0.2) [59]. The tRNAscan-SE (version 2.0.9) algorithm was used to predict tRNA genes [61] and the miRNAs were identified by searching miRBase [62]. Within the Rfamdatabase (version 12.0) [63], identification of the rRNA genes was conducted, and the snoRNA and snRNA genes were also predicted using INFERNAL (version 1.1.1) [64] against the Rfam database (version 14.5) [63]. Pseudogenes were predicted using GenBlastA (version 1.0.4) [65] and GeneWise (version 2.4.1) [66]. GenBlastA was used to scan the whole genomes after masking predicted functional genes [65], and the putative candidates were then analyzed by searching for premature stop codons and frame-shift mutations using GeneWise [66].

The predicted genes were annotated by searching the GenBank Non-Redundant (NR, 20200921, ftp://ftp.ncbi.nlm.nih.gov/blast/db), EggNOG (5.0, http://eggnog5.embl.de/download/eggnog_5.0/) [67], TrEMBL (202005, https://www.uniprot.org), SWISS-PROT (202005, http://ftp.ebi.ac.uk/pub/databases/swissprot) [68], eukaryotic orthologous groups (KOG, 20110125), gene ontology (GO, 20200615, http://geneontology.org) and Kyoto Encyclopedia of Genes and Genomes (KEGG, 20191220, http://www.genome.jp/kegg; 25), and Pfam (version33.1) (http://pfam.xfam.org) [69] databases.

### Transposable element and tandem repeat annotation

Transposable elements (TEs) were identified by a combination of homology-based and de novo approaches. We first customized a de novo repeat library of the genome using RepeatModeler (version 2.0.1) [70], RECON (version 1.08) [71] and RepeatScout (version 1.05) [72]. Then full-length long terminal repeat retrotransposons (fl-LTR-RTs) were identified using both LTRharvest (version 1.5.9) [73] with the parameters:-minlenltr 100 -maxlenltr 40,000 -mintsd 4 -maxtsd 6 -motif TGCA -motifmis 1 -similar 85 -vic 10 -seed 20 -seqids yes and LTR_finder (version 1.0) [74] with the parameters -D 40,000 -d 100 -L 9000 -l 50 -p 20 -C -M 0.9. The high-quality intact fl-LTR-RTs and non-redundant LTR libraries were then produced by LTR_retriever (version 2.8) [75]. A non-redundant species-specific TE library was constructed by combining the de novo TE sequence library above with the Repbase (version 19.06) [76], REXdb (version 3.0) [77], and Dfam (version 3.2) [78] databases. Final TE sequences in the given genomes were identified and classified by a homology search against the library using RepeatMasker (version 4.10). Tandem repeats were annotated by MIcroSAtellite identification tool (MISA) (version 2.1) [79], and Tandem Repeats Finder (version 4.09) [80] with the parameters: 1 1 2 80 5 200 2000 –d -h. We also investigated the correlation between genome size and protein-coding gene numbers as well as the correlation between genome size and the proportion of repetitive elements in the genome based on data on 11 anuran genomes (10 previously published genomes and our newly assembled genome).

### Gene family identification

To identify putative paralogous and orthologous gene clusters, proteins from the longest transcripts of genes from *R. kukunoris, Rhinatrema bivittatum*, *B. gargarizans*, *N. parkeri*, *R. catesbeianus*, *R. marina*, *R. temporaria*, *X. laevis*, *X. tropicalis, V. ailaonica, Microcaecilia Unicolor* and *D. rerio* were compared using Orthofinder software (version 2.4.0) [81] and Diamond was used to align these protein sequences with an e-value of 0.001. The PANTHER database (version 14.0) was used to annotate the obtained gene families [82].

To resolve phylogenetic relationships among *R. kukunoris* and other amphibian species including *R. bivittatum*, *B. gargarizans*, *N. parkeri*, *R. catesbeianus*, *R. marina*, *R. temporaria*, *X. laevis*, *X. tropicalis, V. ailaonica, M. unicolor*, protein sequences from 176 *R. kukunoris* single-copy ortholog genes were used for phylogenetic tree reconstruction, with *D. rerio* used as the outgroup. The protein sequences of the single-copy ortholog genes were aligned with the program mafft (version 5) with the parameters –localpair –maxiterate 1000 [83] and protein alignment was transformed with codon alignment with PAL2NAL (version 14) [84]. The sequence regions with poor alignments were removed using Gblocks (version 0.91b) with the parameters: -b5 = h [85]. After this, all the corresponding coding DNA Sequences (CDS) were concatenated. The best model (GTR + F + I + G4) was determined with ModelFinder [86], and IQtree (version 1.6.11) was used to construct the Maximum Likelihood phylogenetic tree [87] with bootstrap sets of 1000 and GTR + F + I + G4 model.

Divergence times were estimated using MCMCTREE in PAML (version 4.9i) with the model JC69 and the correlated molecular clock [88]. The two parameters (gradient and hessian) were estimated using MCMCTREE. The consistency of the two repeated calculations was 1, and iteration parameters of a Markov chain were -burnin 5,000,000 -sampfreq 30 -nsample 10,000,000. The divergence times were calibrated with estimates from timetree (http://www.timetree.org/) including data for *R. kukunoris* and *R. bivittatum* (311–330 Ma), *X. laevis* and *R. temporaria* (193–223 Ma), and *R. kukunoris* and *R. marina* (145–160 Ma). MCMCTreeR (version 1.1) was used for graphical presentation [89].

To provide more insight into the evolutionary dynamics of the genes, based on the identified gene families and the constructed phylogenetic tree with the predicted divergence times among species, we used CAFE (version 4.2) [90] to analyze gene family expansion and contraction. In CAFE, a random birth and death process was adopted to study gene gain or loss across a specified phylogenetic tree. The gene families with both family-wide and viterbi *p*-values less than 0.05 were considered to have an accelerated rate for gene gain or loss. Identified gene family expansions and contractions in *R. kukunoris* were mapped to KEGG pathways for functional enrichment analysis, which was conducted using the enrichment methods implementing hypergeometric test algorithms and the Q-value (FDR, False Discovery Rate) was calculated to adjust the *p*-values using R-package qvalue [91].

Several genes (*e.g.,* 4-hydroxyphenylpyruvate dioxygenase(hpd), Alkaline Phosphatase(ALPL), Aldehyde dehydrogenase 6 family member A1(Aldh6a1), Glucose-6-phosphate dehydrogenase(G6pd), Phosphoenolpyruvate Carboxykinase 2(PCK2), NAD synthetase 1(NADSYN1), aldehyde dehydrogenase 6 family member A1 (Aldh6a1), endothelial PAS domain protein 1(EPAS1), Flap structure-specific endonuclease 1 (FEN1) and Glucose-6-phosphate dehydrogenase(G6PD) have been shown to be positively selected in high-altitude environments[5, 33, 35, 36]. In order to test whether any of these genes are important in adaptation to high-altitudes in *R. kukunoris*, we identified genes showing signals of positive selection in the *R. kukunoris* genome. Single-copy genes of *N. parkeri*, *R. catesbeiana*, *R. kukunoris*, *R. temporaria*, *B. gargarizans*, and *R. marina* were obtained using Orthofinder (version 2.4). then MAFFT (parameters:–localpair –maxiterate 1000) was used for protein alignment of each gene family, and the protein alignment was transformed with codon alignment in PAL2NAL (version 14) after which the CodeML program in PAML (version 4.9i; F3 × 4 model of codon frequencies) was used to detect positively selected genes in the clade containing *N. parkeri*, *R. catesbeiana*, *R. kukunoris*, *R. temporaria*, *B. gargarizans*, and *R. marina*. Among them, the branch-site model was used, and likelihood ratio tests (LRTs) were calculated (*p* < 0.05) between Model A (foreground clade ω > 1) and null Model (any sites forbidden ω > 1). Posterior probability was calculated using empirical Bayes method (BEB) and posterior probability > 0.95 was considered as positively selected gene. Finally, the R-package clusterProfile was used for the GO and KEGG enrichment analysis of positively selected genes [92].

## Supplementary Information

**Additional file 1.** Supplemental Information.

## Data Availability

All sequence data and assembled scaffolds are deposited in NCBI with BioProject accession number PRJNA787055. All data using this study are deposited in the Additional file 1.
