# Supplementary material for: Chromosome-level genome assembly of a high-altitude-adapted frog (Rana kukunoris) from the Tibetan plateau provides insight into amphibian genome evolution and adaptation
Source: Front Zool. 2023 Jan 6;20:1. doi: 10.1186/s12983-022-00482-9 (PMC9817415; doi:10.1186/s12983-022-00482-9)

**Additional file 1.**

**Supplemental Information for:**

**Chromosome-level Genome Assembly of** **a high-altitude-adapted frog (*Rana kukunoris*) from the Tibetan plateau provides insight into** **amphibian genome evolution and adaptation**

Wei Chen^1,2,3^*, Hongzhou Chen^1^, Jiahong Liao^4^, Min Tang^4^, Haifen Qin^4^, Zhenkun Zhao^4^,Xueyan Liu^1^, Yanfang Wu^1^, Lichun Jiang^4^, Lixia Zhang^5^, Bohao Fang^6^, Xueyun Feng^7^, Baowei Zhang^8^, Kerry Reid^9^ & Juha Merilä^7,9^

^1^School of Resources and Environmental Engineering, Anhui University, Hefei 230601, China

^2^Anhui Shengjin Lake Wetland Ecology National Long-term Scientific Research Base, Dongzhi, 247230, China

^3^Anhui Province Key Laboratory of Wetland Ecosystem Protection and Restoration, Anhui University, Hefei 230601, China

^4^School of Life Science and Technology, Mianyang Normal University, Mianyang, Sichuan 621000, China

^5^Department of Ecology, College of Life Sciences, Henan Normal University, Xinxiang 453007, China

^6^Department of Organismic and Evolutionary Biology and Museum of Comparative Zoology, Harvard University, 26 Oxford Street, Cambridge, MA, USA.

^7^Ecological Genetics Research Unit, Research Programme in Organismal and Evolutionary Biology, Faculty of Biological and Environmental Sciences, University of Helsinki, Helsinki 00014, Finland

^8^School of Life Sciences, Anhui University, Hefei, 230601,China

^9^Area of Ecology and Biodiversity, School of Biological Sciences, The University of Hong Kong, Hong Kong SAR, China

* corresponding author: wchen1949@163.com

**Table of Contents:**

| Table S1 | The websites for downloading the genomic data of other species in this study | Page 3 |
| --- | --- | --- |
| Table S2 | K-mer data statistics | Page 3 |
| Table S3 | Summary of [PacBio sequencing](https://www.so.com/link?m=bfHVn9MjkiBF1AmNAWWWoSsOcNK1b08Q8usAq7Gfze1qJwYlnm6oJYelT23qZwPOqug2GTRS5zdYCl7FCO+gd3V23ineQWUoU+jcjGCDJ7jcFnyUA81HCbXEw5WUgZ9J/CRTEAHxgcnDWLsNHoYGG2hXVZJQ=) data | Page 3 |
| Table S4 | The length distribution of PacBio reads | Page 3 |
| Table S5 | The statistics of genome assembly | Page 4 |
| Table S6 | Genome assembly statistics for published amphibian genome | Page4 |
| Table S7 | Information on 12 *Rana kukunoris* chromosomes after Hi-C assembly | Page 4 |
| Table S8 | Statistical mapping results of Illumina sequencing data | Page 5 |
| Table S9 | Genome completeness of the *Rana kukunoris* genome assembly as evaluated by BUSCO statistics. | Page 5 |
| Table S10 | Statistical analysis of Gene function annotation | Page 5 |
| Table S11 | Descriptive statistics of genes in different amphibian genomes | Page 5 |
| Table S12 | Total number of different types repeat elements in the *Rana kukunoris* genome assembly. Given are total number of elements of each type, their total length, and proportion of the total genome they account for. | Page6-7 |
| Table S13 | Results of gene ontology analyses for the top 30 expanded genes families | Page 7 |
| Table S14 | The genes in the *Rana kukunoris* genome identified to have been under positive selection | Page 8-10 |
| Fig. S1 | KEGG pathway enrichment analysis of positively selected genes | Page 11-12 |

**Table S1** The websites for downloading the genomic data of other species in this study

| **species** | **Download** |
| --- | --- |
| *Bufo gargarizans* | https://www.ncbi.nlm.nih.gov/genome/?term=Bufo_gargarizans |
| *Danio rerio* | ftp://ftp.ncbi.nlm.nih.gov/genomes/all/GCF/000/002/035/GCF_000002035.6_GRCz11/GCF_000002035.6_GRCz11_genomic.fna.gz |
| *Microcaecilia unicolor* | https://www.ncbi.nlm.nih.gov/genome/?term=Microcaecilia+unicolor |
| *Nanorana parkeri* | https://www.ncbi.nlm.nih.gov/genome/?term=Nanorana+parkeri |
| *Rana catesbeiana* | https://www.ncbi.nlm.nih.gov/genome/?term=Rana+catesbeianus |
| *Rana kukunoris* | this study |
| *Rana temporaria* | https://www.ncbi.nlm.nih.gov/genome/?term=Rana+temporaria |
| *Rhinatrema bivittatum* | https://www.ncbi.nlm.nih.gov/genome/?term=Rhinatrema+bivittatum |
| *Rhinella marina* | https://ftp.cngb.org/pub/gigadb/pub/10.5524/100001_101000/100483/canetoad.v2.2.fasta.gz |
| *Vibrissaphora ailaonica* | http://gigadb.org/dataset/100624 |
| *Xenopus laevis* | https://www.ncbi.nlm.nih.gov/genome/?term=Xenopus+laevis |
| *Xenopus tropicalis* | https://www.ncbi.nlm.nih.gov/genome/?term=Xenopus+tropicalis |

Table S2. K-mer data statistics

| **Name** | **K-mer** | **K-mer Depth** | **Genome size (bp)** | **Heterozygous ratio (%)** |
| --- | --- | --- | --- | --- |
| ***Rana kukunoris*** | 21 | 29 | 4,489,353,974 | 0.3% |

**Table S3** Summary of PacBio sequencing data

| Data_Type | Reads_number | Reads_base | N50_Reads_Length | Mean_ Reads_Length | Max_Reads_Length |
| --- | --- | --- | --- | --- | --- |
| Subreads | 21,322,047 | 437,678,042,147 | 30,654 | 20,527 | 500,865 |
| ZMWreads | 18,496,760 | 384,781,293,680 | 31,852 | 20,803 | 500,865 |

**Table S4** The length distribution of PacBio reads

| Length | Total number | Total length | Average length |
| --- | --- | --- | --- |
| 500~2000 | 1,423,716 | 1,752,168,300 | 1,230.70 |
| 2000~4000 | 1,641,562 | 4,882,683,782 | 2,974.41 |
| 4000~6000 | 1,450,259 | 7,227,491,523 | 4,983.59 |
| 6000~8000 | 1,322,596 | 9,238,863,012 | 6,985.40 |
| 8000~10000 | 1,221,592 | 10,978,578,532 | 8,987.11 |
| 10000~12000 | 1,136,070 | 12,481,639,851 | 10,986.68 |
| 12000~14000 | 1,063,536 | 13,814,201,085 | 12,988.94 |
| 14000~16000 | 1,002,658 | 15,030,638,787 | 14,990.79 |
| 16000~18000 | 966,719 | 16,429,963,210 | 16,995.59 |
| 18000~ | 10,093,339 | 345,841,814,065 | 34,264.36 |

**Table S5** The statistics of genome assembly with PacBio sequencing data

| Contig number | Total contig length(bp) | Contig N50(bp) | Contig N90(bp) | Contig max (bp) | GC content(%) | Gap total length(bp) |
| --- | --- | --- | --- | --- | --- | --- |
| 6,393 | 4,830,373,361 | 1,798,518 | 378,599 | 11,379,543 | 44.41 | 0 |

**Table S6** Genome assembly statistics for published anura genomes

|  | *Xenopus tropicalis* | *Nanorana parkeri* | *Xenopus laevis* | *Rhinella marina* | *Rana*  *catesbeianus* | *Oophaga pumilio* | *Leptobrachium leishanense* | *Vibrissaphora ailaonica* | *Bufo gargarizans* | *Rana temporaria* | *Rana*  *kukunoris* |
| --- | --- | --- | --- | --- | --- | --- | --- | --- | --- | --- | --- |
| Size (Gb) | 1.51 | 2.05 | 2.74 | 2.55 | 5.8 | 5.5 | 3.55 | 3.53 | 4.55 | 4.11 | 4.83 |
| coverage (×) | 111.5 | 83 | 28.5 | 141 | 66.0 | 136.0 | 80.3 | 78.0 | 103.0 | 63 | 90.6 |
| Contig N50 (bp) | 71,041 | 32,920 | 19,713 | 167,489 | 5,239 | 3,942 | 1,931,129 | 821,125 | 1,738,317 | 27,000 | 1,798,518 |
| Scaffobpld N50(bp) | 135,134,832 | 1,075,367 | 136,570,856 | 167,489 | 68,964 | 59,503 | 394,693,044 | 412.42mb | 539.82mb | 482mb | 547,819,331 |
| GC content (%) | 40.53 | 42.5 | 39.3 | 43.2 | 43.0 | 43.5 | 43.75 | 43.68 | 44.4 | 44.19 | 43.62 |
| Protein coding genes | 27,047 | 37，385 | 37,385 | 58,302 | [22,0](https://www.ncbi.nlm.nih.gov/genome/browse/#!/proteins/23031/353399\|Lithobates%20catesbeianus/)00 | 17,051 | 23,420 | 26,227 | 36,698 | 23,707 | 32,304 |
| **Repetitive sequences（%）** | 51.9 | 56.7 | 56.7 | 86.6 | 66.9 | 62.9 | 77.1 | 69.5 | 65.61 | - | 70.9 |
| Assembly level | scaffolds | Scaffold | Chromosome | Contig | Scaffold | Scaffold | Chromosome | Chromosome | Chromosome | Chromosome | Chromosome |
| Date | 2019 | 2015 | 2016 | 2018 | 2017 | 2019 | 2019 | 2019 | 2020 | 2022 | 2023 |
| **References** | Hellsten et al., 2011 | [Sun](https://www.webofscience.com/wos/alldb/general-summary?queryJson=%5b%7b) et al., 2015 | Session et al.,2016 | Edwards et al., 2018 | [Hammond](https://pubmed.ncbi.nlm.nih.gov/?term=Hammond+SA&cauthor_id=29127278) et al.,2017 | Li, et al., 2019b | Li, et al., 2019b | Li et al., 2019a | Lu et al., 2021 | Streicher et al., 2022 | This study |

**Table S7** Information on 12 *R. kukunoris* chromosomes after Hi-C assembly.

| Chromosome | **Cluster Number** | **Cluster Length (bp)** | **Order Number**** | **Order Length ***(bp)** |
| --- | --- | --- | --- | --- |
| Chr01 | 1451 | 816339647 | 788 | 745207392 |
| Chr02 | 754 | 648941533 | 462 | 616183699 |
| Chr03 | 629 | 574074147 | 403 | 547779131 |
| Chr04 | 670 | 590189449 | 419 | 561369042 |
| Chr05 | 544 | 508592439 | 327 | 483268977 |
| Chr06 | 332 | 264505781 | 182 | 247596571 |
| Chr07 | 347 | 249680029 | 172 | 227884912 |
| Chr08 | 289 | 214480542 | 143 | 188545849 |
| Chr09 | 345 | 234623573 | 165 | 212835008 |
| Chr10 | 281 | 178939182 | 142 | 161726670 |
| Chr11 | 501 | 360053305 | 245 | 329732986 |
| Chr12 | 262 | 173926295 | 136 | 158354105 |
| Total(Ratio* %) | 6405(97.71) | 4814345922(99.67) | 3584(55.96) | 4480484342(93.07) |

*The ratio calculated only using sequences longer than 1Kb and not including 100 Ns added!

**The number of sequences whose order and direction along a particular chromosome was determined

***The length of the sequences whose order and direction along a particular chromosome was determined

**Table S8** Mapping results of Illumina sequencing data back to genome assembly based on PacBio sequencing data

| Total Reads | Mapped Reads | Mapping Rate (%) | Properly Paired Reads | Proper Map Rate (%) |
| --- | --- | --- | --- | --- |
| 2,160,510,068 | 2,149,747,888 | 99.5 | 2,115,826,610 | 97.93 |

| Complete BUSCOs | Complete and single-copy BUSCOs | Complete and duplicated BUSCOs | Fragmented BUSCOs | Missing BUSCOs | Total Lineage BUSCOs |
| --- | --- | --- | --- | --- | --- |
| 3096(92.31%) | 2945 (87.81%) | 151 (4.50%) | 63 (1.88%) | 195 (5.81%) | 3,354 |

**Table S****9** Genome completeness of the *R. kukunoris* genome assembly as evaluated by BUSCO statistics.

**Table S10** Statistics for Gene function annotation

| Annotation_Database | Annotated_Number | Annotated_Ratio(%) |
| --- | --- | --- |
| GO_Annotation | 24559 | 76.02 |
| KEGG_Annotation | 28388 | 87.88 |
| KOG_Annotation | 19482 | 60.31 |
| Pfam_Annotation | 29107 | 90.1 |
| Swissprot_Annotation | 26906 | 83.29 |
| TrEMBL_Annotation | 31816 | 98.49 |
| EggNOG_Annotation | 27077 | 83.82 |
| NR_Annotation | 31905 | 98.76 |
| All_Annotated | 31957 | 98.93 |
| total_genes | 32,304 |  |

**Table S11** Descriptive statistics of genes in different amphibian genomes

| Species | Gene_  Number | Gene_  Length  (bp) | Average_  Gene_  Length  (bp) | Exon_  Length  (bp) | Average_Exon_  Length  (bp) | Exon_  Number | Average_Exon_  Number | CDS_  Length  (bp) | Average_  CDS_  Length  (bp) | CDS_  Number | Average_CDS_  Number | Intron_  Length  (bp) | Average_  Intron_  Length(bp) | Intron_  Number | Average_Intron_  Number |
| --- | --- | --- | --- | --- | --- | --- | --- | --- | --- | --- | --- | --- | --- | --- | --- |
| *R. temporaria* | 23570 | 1861883782 | 78993.8 | 59612292 | 2529.16 | 224604 | 9.53 | 40129837 | 1702.58 | 210326 | 8.92 | 1802271490 | 76464.64 | 200739 | 8.52 |
| *N. parkeri* | 18564 | 840040735 | 45251.06 | 35282578 | 1900.59 | 184048 | 9.91 | 30667010 | 1651.96 | 178579 | 9.62 | 804758157 | 43350.47 | 165309 | 8.9 |
| *X. laevis* | 31182 | 1090803135 | 34981.82 | 85421582 | 2739.45 | 323441 | 10.37 | 52751399 | 1691.73 | 302185 | 9.69 | 1005381553 | 32242.37 | 292033 | 9.37 |
| *B. bufo* | 21324 | 1660263176 | 77858.9 | 53395504 | 2504.01 | 221385 | 10.38 | 35897586 | 1683.44 | 202216 | 9.48 | 1606867672 | 75354.89 | 199931 | 9.38 |
| *R. marina* | 58302 | 1097619455 | 18826.45 | 72282700 | 1239.8 | 309718 | 5.31 | 60829383 | 1043.35 | 294535 | 5.05 | 1025336755 | 17586.65 | 251416 | 4.31 |
| *R. catesbeianus* | 22201 | 186808794 | 8414.43 | 30074814 | 1354.66 | 92710 | 4.18 | 16498161 | 743.13 | 88130 | 3.97 | 156733980 | 7059.77 | 70509 | 3.18 |
| *R. kukunoris* | 32304 | 1736981706 | 53769.86 | 78491340 | 2429.77 | 232318 | 7.19 | 56045371 | 1734.94 | 225422 | 6.98 | 1658490366 | 51340.09 | 200014 | 6.19 |
| *X. tropicalis* | 21715 | 733824683 | 33793.45 | 64051628 | 2949.65 | 231138 | 10.64 | 37208934 | 1713.51 | 211115 | 9.72 | 669773055 | 30843.8 | 209256 | 9.64 |

**Table S12** Total number of different types repeat elements in the *Rana kukunoris* genome assembly. Given are total number of elements of each type, their total length, and proportion of the total genome they account for (Rate (%)).

| **Type** | **Number** | **Length** | **Rate(%)** |
| --- | --- | --- | --- |
| ClassI:Retroelement | 5,945,738 | 1,864,828,457 | 38.6 |
| ClassI/DIRS | 215,417 | 135,912,968 | 2.81 |
| ClassI/LINE | 870,425 | 304,655,012 | 6.31 |
| ClassI/LTR/Cassandra | 8 | 420 | 0 |
| ClassI/LTR/Caulimovirus | 117 | 7,202 | 0 |
| ClassI/LTR/Copia | 251,290 | 60,367,722 | 1.25 |
| ClassI/LTR/ERV | 112,133 | 16,993,480 | 0.35 |
| ClassI/LTR/Gypsy | 1,594,734 | 547,644,180 | 11.34 |
| ClassI/LTR/Pao | 38,927 | 8,029,174 | 0.17 |
| ClassI/LTR/Unknown | 2,747,680 | 765,602,706 | 15.85 |
| ClassI/LTR/Viper | 136 | 13,715 | 0 |
| ClassI/SINE | 114,871 | 25,601,878 | 0.53 |
| ClassII:DNA transposon | 4,003,634 | 1,103,980,595 | 22.85 |
| ClassII/Academ | 578 | 73,614 | 0 |
| ClassII/CACTA | 149,821 | 27,707,312 | 0.57 |
| ClassII/Crypton | 6,901 | 1,000,940 | 0.02 |
| ClassII/Dada | 19,927 | 6,409,085 | 0.13 |
| ClassII/Ginger | 3,439 | 605,412 | 0.01 |
| ClassII/Helitron | 17,409 | 4,215,165 | 0.09 |
| ClassII/IS3EU | 6,264 | 1,371,149 | 0.03 |
| ClassII/Kolobok | 7,280 | 705,320 | 0.01 |
| ClassII/MITE | 109 | 6,440 | 0 |
| ClassII/Maverick | 30,863 | 5,098,614 | 0.11 |
| ClassII/Merlin | 1,175 | 84,933 | 0 |
| ClassII/Mutator | 39,942 | 6,078,939 | 0.13 |
| ClassII/Novosib | 3,655 | 557,535 | 0.01 |
| ClassII/P | 4,634 | 419,632 | 0.01 |
| ClassII/PIF-Harbinger | 176,908 | 118,073,628 | 2.44 |
| ClassII/PiggyBac | 462,509 | 180,105,637 | 3.73 |
| ClassII/Sola | 2,997 | 506,571 | 0.01 |
| ClassII/Tc1-Mariner | 728,855 | 216,887,893 | 4.49 |
| ClassII/Unknown | 1,322,404 | 271,155,226 | 5.61 |
| ClassII/Zator | 454 | 39,452 | 0 |
| ClassII/Zisupton | 5,776 | 658,413 | 0.01 |
| ClassII/hAT | 1,011,734 | 262,219,685 | 5.43 |
| srpRNA | 2 | 241 | 0 |
| Total | 9,949,374 | 2,968,809,293 | 61.46 |

**Table S13** Results of gene ontology analyses for the top 30 expanded genes families

| **GO ID** | **GO Name** | **GO Category** | ***p*-value** | **q-value** |
| --- | --- | --- | --- | --- |
| GO:0004190 | MF | aspartic-type endopeptidase activity | 9.90E-87 | 7.50E-85 |
| GO:0005637 | CC | nuclear inner membrane | 3.69E-37 | 2.37E-35 |
| GO:0004523 | MF | RNA-DNA hybrid ribonuclease activity | 1.23E-34 | 4.68E-33 |
| GO:0019866 | CC | organelle inner membrane | 1.63E-31 | 5.23E-30 |
| GO:0031965 | CC | nuclear membrane | 6.77E-31 | 1.45E-29 |
| GO:0005635 | CC | nuclear envelope | 2.99E-27 | 4.79E-26 |
| GO:0000785 | CC | chromatin | 1.34E-25 | 1.72E-24 |
| GO:0005521 | MF | lamin binding | 2.08E-22 | 4.36E-21 |
| GO:0008527 | MF | taste receptor activity | 2.30E-22 | 4.36E-21 |
| GO:0031967 | CC | organelle envelope | 1.85E-20 | 1.70E-19 |
| GO:0031975 | CC | envelope | 1.85E-20 | 1.70E-19 |
| GO:0044427 | CC | obsolete chromosomal part | 4.26E-20 | 3.42E-19 |
| GO:0033038 | MF | bitter taste receptor activity | 2.24E-19 | 3.40E-18 |
| GO:0050912 | BP | detection of chemical stimulus involved in sensory perception of taste | 2.26E-18 | 3.27E-16 |
| GO:0050913 | BP | sensory perception of bitter taste | 2.26E-18 | 3.27E-16 |
| GO:0001580 | BP | detection of chemical stimulus involved in sensory perception of bitter taste | 4.63E-18 | 5.58E-16 |
| GO:0006508 | BP | proteolysis | 9.76E-18 | 1.01E-15 |
| GO:0042626 | MF | ATPase-coupled transmembrane transporter activity | 1.22E-17 | 1.54E-16 |
| GO:0005694 | CC | chromosome | 2.11E-17 | 1.51E-16 |
| GO:0051607 | BP | defense response to virus | 2.28E-17 | 2.06E-15 |
| GO:0007606 | BP | sensory perception of chemical stimulus | 4.44E-17 | 3.57E-15 |
| GO:0098542 | BP | defense response to other organism | 1.71E-16 | 1.23E-14 |
| GO:0009615 | BP | response to virus | 3.13E-16 | 1.90E-14 |
| GO:0050906 | BP | detection of stimulus involved in sensory percep..\|detection of stimulus involved in sensory perception | 3.15E-16 | 1.90E-14 |
| GO:0045095 | CC | keratin filament | 1.01E-15 | 6.49E-15 |
| GO:0050909 | BP | sensory perception of taste | 2.22E-14 | 1.23E-12 |
| GO:0032367 | BP | intracellular cholesterol transport | 4.22E-12 | 2.18E-10 |
| GO:0051606 | BP | detection of stimulus | 1.17E-11 | 5.62E-10 |
| GO:0006952 | BP | defense response | 1.36E-11 | 6.16E-10 |
| GO:0016459 | CC | myosin complex | 4.07E-10 | 2.38E-09 |

*****Abbreviations: BP: Biological process; CC: Cellular component; MF: Molecular function

**Table S14** The genes in the *Rana kukunoris* genome identified to have been under positive selection

| #Gene | Branch_p_value | Gene_family |
| --- | --- | --- |
| Rk01G017720 | 0.000000297 | OG0001299 |
| Rk03G023960 | 0.028627714 | OG0001486 |
| Rk0G018470 | 0.048610827 | OG0001852 |
| Rk01G021660 | 0 | OG0001860 |
| Rk04G006330 | 0 | OG0002125 |
| Rk02G014850 | 0 | OG0002155 |
| Rk0G013550 | 0 | OG0002255 |
| Rk03G006270 | 0.000120717 | OG0002495 |
| Rk12G002880 | 0 | OG0002552 |
| Rk0G009970 | 0 | OG0002555 |
| Rk01G034130 | 0.000010375 | OG0002600 |
| Rk03G023510 | 0 | OG0002639 |
| Rk01G039080 | 0.000145034 | OG0002707 |
| Rk01G017570 | 0 | OG0002772 |
| Rk03G025920 | 0 | OG0002780 |
| Rk0G000050 | 0.011091869 | OG0002958 |
| Rk01G033030 | 0.000618867 | OG0003030 |
| Rk01G033890 | 0 | OG0003073 |
| Rk03G022400 | 0.009162704 | OG0003142 |
| Rk02G034220 | 0.000146696 | OG0003188 |
| Rk12G010470 | 0 | OG0003437 |
| Rk03G026500 | 0 | OG0003451 |
| Rk01G022270 | 0 | OG0003461 |
| Rk12G001560 | 0.000211039 | OG0003502 |
| Rk01G036270 | 0.000000001 | OG0003545 |
| Rk0G013370 | 0 | OG0003588 |
| Rk11G021790 | 0.007451239 | OG0003920 |
| Rk11G019760 | 0.000000079 | OG0003986 |
| Rk04G009130 | 0.000003748 | OG0003996 |
| Rk02G020220 | 0.000034417 | OG0004110 |
| Rk01G003660 | 0.017314621 | OG0004187 |
| Rk09G005640 | 0.000000001 | OG0004205 |
| Rk02G004510 | 0.000147353 | OG0004337 |
| Rk03G023650 | 0.00000004 | OG0004469 |
| Rk06G011600 | 0.048326172 | OG0004476 |
| Rk06G017760 | 0.000093234 | OG0004543 |
| Rk03G023360 | 0.000050189 | OG0004601 |
| Rk03G013760 | 0.000001062 | OG0004692 |
| Rk01G019690 | 0 | OG0005059 |
| Rk01G012850 | 0 | OG0005113 |
| Rk07G007130 | 0.0219102 | OG0005133 |
| Rk0G017130 | 0.005003293 | OG0005191 |
| Rk02G009080 | 0.000022701 | OG0005211 |
| Rk03G011910 | 0.001576978 | OG0005487 |
| Rk04G027750 | 0 | OG0005491 |
| Rk0G027070 | 0 | OG0005574 |
| Rk04G031410 | 0.002300391 | OG0005656 |
| Rk11G014690 | 0.00000003 | OG0005724 |
| Rk10G003440 | 0 | OG0005956 |
| Rk03G032480 | 0 | OG0006085 |
| Rk04G009550 | 0.00100274 | OG0006343 |
| Rk11G014600 | 0.000000255 | OG0006381 |
| Rk01G034530 | 0.000030616 | OG0006456 |
| Rk05G021230 | 0 | OG0006488 |
| Rk04G010830 | 0.020782521 | OG0006517 |
| Rk04G029560 | 0 | OG0006660 |
| Rk03G005470 | 0.016517332 | OG0006679 |
| Rk11G001290 | 0.000000001 | OG0006697 |
| Rk0G000500 | 0.003935131 | OG0006897 |
| Rk11G014290 | 0.000025297 | OG0007093 |
| Rk05G015430 | 0.00090215 | OG0007166 |
| Rk02G029490 | 0.000001592 | OG0007424 |
| Rk03G000760 | 0.000003539 | OG0007438 |
| Rk04G005790 | 0 | OG0007567 |
| Rk04G018350 | 0.02001979 | OG0007688 |
| Rk03G010230 | 0.020655472 | OG0007757 |
| Rk02G006210 | 0 | OG0007778 |
| Rk12G003240 | 0.000140142 | OG0007885 |
| Rk01G005620 | 0.000000183 | OG0007948 |
| Rk11G012010 | 0 | OG0008013 |
| Rk03G029130 | 0.04261811 | OG0008153 |
| Rk12G004330 | 0.017926719 | OG0008171 |
| Rk09G014410 | 0.000000001 | OG0008912 |
| Rk03G011440 | 0.028443318 | OG0009099 |
| Rk01G031840 | 0 | OG0009225 |
| Rk02G030650 | 0.032486554 | OG0009499 |
| Rk05G017510 | 0 | OG0009698 |
| Rk04G022200 | 0.000000023 | OG0009804 |
| Rk11G012870 | 0.000003041 | OG0009808 |
| Rk04G014940 | 0 | OG0009924 |
| Rk05G008270 | 0.035276827 | OG0010074 |
| Rk01G011130 | 0.00000004 | OG0010161 |
| Rk12G008670 | 0 | OG0010314 |
| Rk01G030830 | 0 | OG0010326 |
| Rk05G015260 | 0.00000121 | OG0010630 |
| Rk02G008110 | 0.026125563 | OG0010664 |
| Rk03G023560 | 0.0000098 | OG0010669 |
| Rk11G005010 | 0 | OG0010824 |
| Rk04G006050 | 0.001305541 | OG0011305 |
| Rk06G014070 | 0 | OG0011437 |
| Rk09G012620 | 0 | OG0011888 |
| Rk04G013440 | 0.000532101 | OG0012010 |
| Rk03G008830 | 0.000015866 | OG0012366 |
| Rk03G011050 | 0 | OG0012523 |
| Rk08G009250 | 0.015013702 | OG0012787 |
| Rk04G023270 | 0.011958978 | OG0013460 |
| Rk01G023560 | 0.000127534 | OG0014188 |

Fig.S1 KEGG pathway enrichment analysis of positively selected genes.


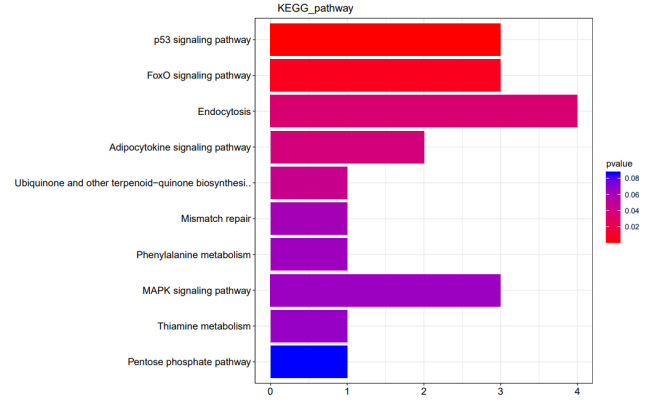

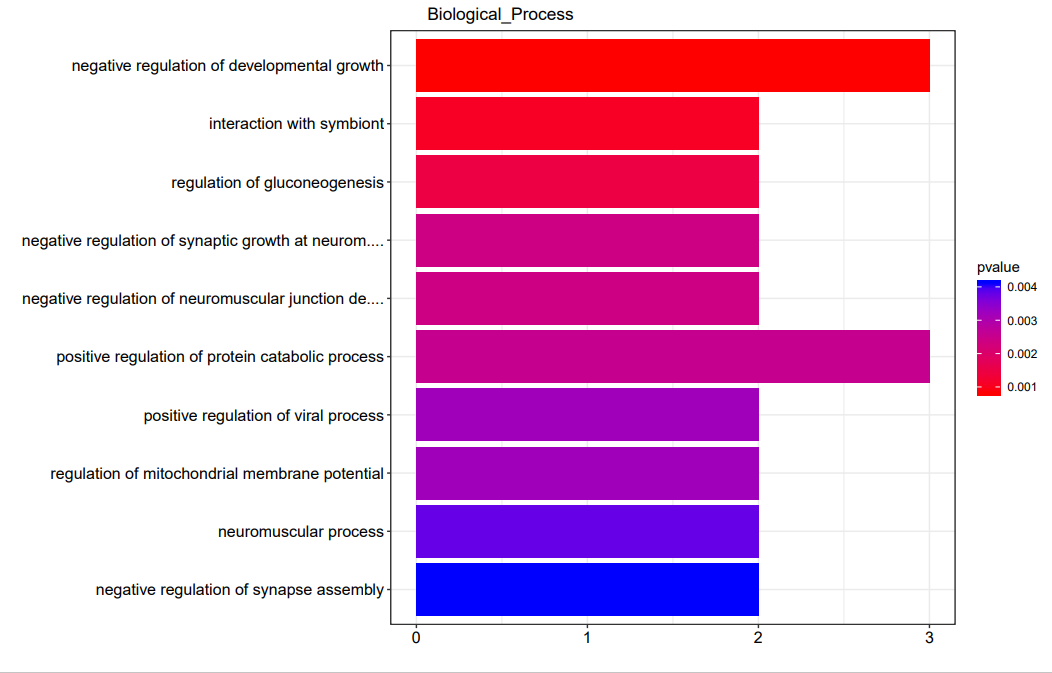


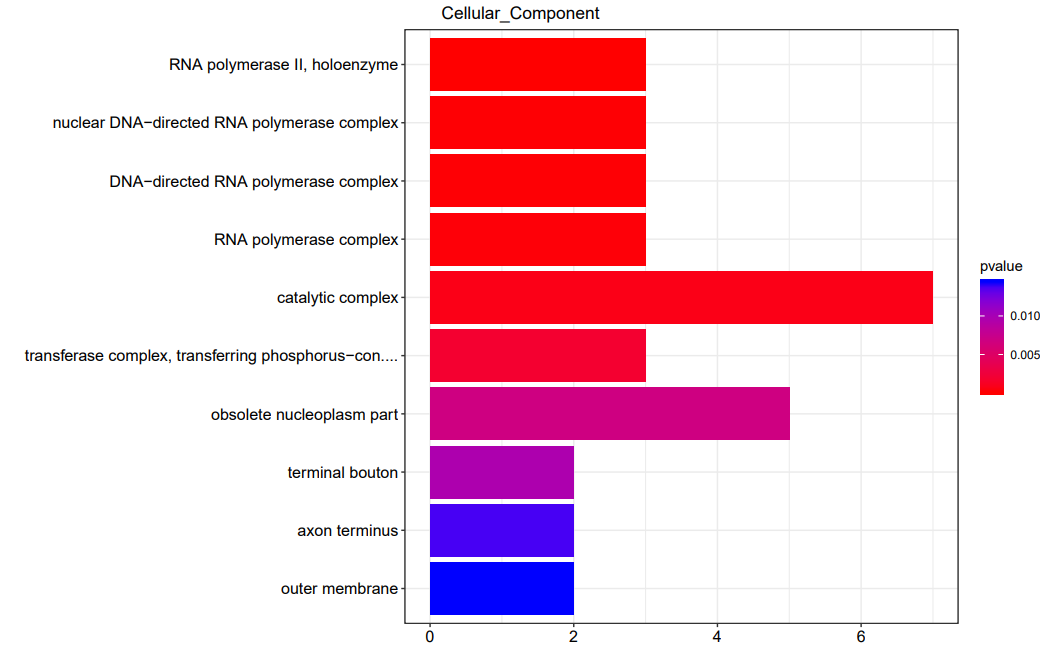

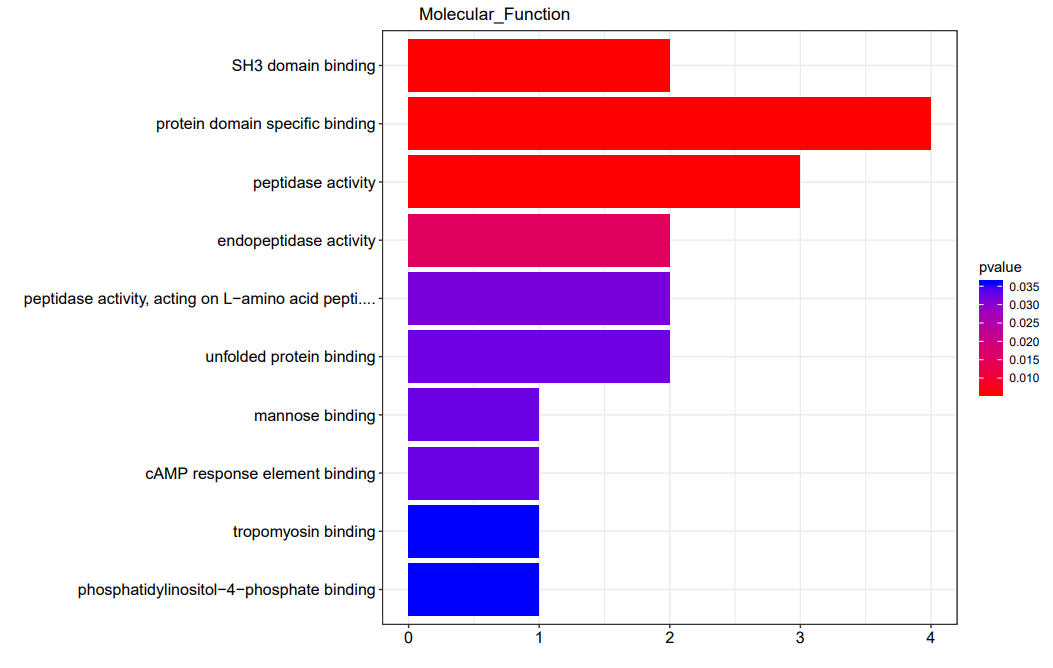

Supplement: Supplementary file 1 — Additional file 1. Supplemental Information. [file 12983_2022_482_MOESM1_ESM.docx]
